# Dual targeting of EZH2 and PD-L1 in Burkitt’s lymphoma enhances immune activation and induces apoptotic pathway

**DOI:** 10.3389/fimmu.2025.1578665

**Published:** 2025-04-16

**Authors:** Yurim Jeong, Hyewon Jang, Se Been Kim, Minseo Yu, Ra Eun Kim, Wan-Su Choi, Youngwoo Jeon, Jung-Yeon Lim

**Affiliations:** ^1^ Department of Biomedical Laboratory Science, Inje University, Gimhae, Republic of Korea; ^2^ Department of Hematology, Yeouido St. Mary Hospital, School of Medicine, The Catholic University of Korea, Seoul, Republic of Korea

**Keywords:** Burkitt’s lymphoma, ehz2, immune checkpoint, anti-PD-1, protac, apoptosis

## Abstract

**Introduction:**

Enhancer of zeste homolog 2 (EZH2) catalyzes H3K27me3, an epigenetic modification linked to gene silencing, and its overexpression contributes to the progression of hematological malignancies. This study compares the efficacy of a conventional EZH2 inhibitor with a PROTAC-based EZH2 degrader in human lymphoma cell lines. Furthermore, we investigate the anti-tumor effects of combining EZH2 degrader with anti-PD-1, an immune checkpoint inhibitor, focusing on immune cell interactions and underlying mechanisms.

**Methods:**

The cytotoxic effects of the EZH2 degrader and EZH2 inhibitor were evaluated in Burkitt’s, B-cell, cutaneous T-cell, and Hodgkin’s lymphoma cell lines. Additionally, the combination therapy of the EZH2 degrader and anti-PD-1 was assessed both *in vitro* and in a hu-PBMC-CDX mouse model.

**Results:**

We evaluated the effects of an EZH2 degrader on seven lymphoma cell lines and observed significant reductions in cell viability compared to EZH2 inhibitor, particularly in Burkitt’s lymphoma cell lines. EZH2 degrader treatment reduced EZH2 and c-Myc expression, induced G2/M cell cycle arrest, and increased apoptosis markers, including cleaved caspase-3 and cleaved PARP. Furthermore, Burkitt’s lymphoma is a PD-L1 negative tumor; however, treatment with the EZH2 degrader resulted in a slight increase in PD-L1 expression. Combining EZH2 degrader with anti-PD-1 significantly enhanced anti-tumor effects compared to monotherapy. *In vivo* studies using a humanized lymphoma mouse model demonstrated a synergistic anti-tumor effect of EZH2 degrader and anti-PD-1, which was attributed to apoptosis-related pathways.

**Discussion:**

These findings aim to provide insights into the therapeutic potential of targeting EZH2 in combination with immune checkpoint inhibitors for improved treatment of lymphomas.

## Introduction

Lymphoma is a group of the most common hematologic malignancies that originates in the lymphocytes, characterized by more than 45 subtypes, including both Hodgkin lymphoma and non-Hodgkin lymphoma. There are more than 600 000 new cases of lymphoma annually, and it ranks as the 10^th^ most commonly diagnosed cancer worldwide. As there are many types of lymphoma, the prognosis also varies, but some aggressive lymphomas have metastases throughout the body and have a poor prognosis, which requires rapid and effective treatment (1, 2).

Enhancer of zeste homolog 2 (EZH2) is a subunit of the polycomb repressive complex 2 (PRC2) and mediates trimethylation of histone H3 lysine 27 (H3K27me3) (3). The suppression of epigenetic gene expression resulting from the overexpression of EZH2 is associated with poor prognosis in various cancers (4, 5). Previous studies have shown that EZH2 is overexpressed in many solid cancers, including breast cancer (6), lung cancer (7), ovarian cancer (8) and colon cancer (9), and is linked to cancer proliferation and aggressiveness. Furthermore, the removal of the PRC2/EZH2 complex in a mouse model of EZH2-overexpressing breast cancer can inhibit cancer proliferation, supporting the notion that EZH2 may influence cancer proliferation (10).

Recent research supports that EZH2 is one of key factors contributing to the pathogenesis of lymphoma. EZH2 mutation, including the mutation of Y641, A687V and A677G, leads to promotion lymphomagenesis in B-cells (11–13). Overexpression of EZH2 has also been confirmed in hematologic malignancies, such as lymphomas, with particularly high expression observed in Burkitt’s lymphoma (14). This finding supports that targeting EZH2 in Burkitt’s lymphoma may represent a potential therapeutic strategy.

EZH2 inhibitors, including Tazemetostat, have shown promising results in clinical trials; however, there are instances of limited efficacy leading to suboptimal therapeutic outcomes (15, 16). PROTAC technology, using the ubiquitin-proteasome system, connects the ligand for the protein of interest (POI) and the ligand of the E3 ubiquitin ligase with a linker and induces the degradation of POI. MS1943, EZH2 degrader utilizing PROTAC technology, has demonstrated remarkable anti-tumor effects in prior studies on breast cancer and Burkitt’s lymphoma (17–19). Therefore, we will investigate the impact of EZH2 on the survival of Burkitt’s lymphoma using Tazemetostat and MS1943.

Additionally, abnormal epigenetic modifications are recognized as essential components of cancer immune evasion (20). Hence, the epigenetic regulation of gene expression by EZH2 is acknowledged as a key factor in modulating immune evasion, such as controlling the expression of PD-L1, in cancer cells (21).

Our study focuses on evaluating the combined efficacy of EZH2 inhibition and the immune checkpoint inhibitor anti-PD-1 specifically in cytotoxic T lymphocytes co-cultured Burkitt’s lymphoma. We also aim to elucidate the mechanism in preclinical models and *in vitro*. In conclusion, our study provides a promising therapeutic strategy to improve lymphoma therapeutic efficacy by blocking immunological evasion that occurs by inhibition of EZH2.

## Methods

### Mice and treatments

Female NOD/SCID/IL2 receptor γ c null (NOG) mice (n=44, 8-week old, Saeronbio) were housed in-group in cages at a mean constant temperature (25 ± 2°C), humidity (55 ± 5%) and illumination (12 h light-dark cycle), and free access to standard pellet chow and water. The mice were kept at the Clinical Medicine Research Institute, Yeouido St. Mary’s Hospital, Catholic University of Korea (approval ID: YEO-2022-006-01T). For humanization, mice were injected intravenously with 1 × 10^7^ human PBMC via the tail vein. Peripheral blood from all mice was monitored for human T cell (CD45+CD3+CD8+) reconstitution at the end of experiment. Human CD45 (#304012), human CD3 (#317308) and human CD8 (#344704) antibodies were purchased from BioLegend (CA, USA). Mice that had over 25% CD45+CD3+ cells in the peripheral blood were considered to constitute the Hu-PBMC model. Cell suspensions were prepared from *in vitro*-cultured Daudi cells. After 5 days from human PBMC inoculation, mice were injected intraperitoneally with 2 × 10^6^ Daudi cells in PBS. Each mouse was treated twice a week with MS1943 at 50 mg/kg (n=11), Pembrolizumab at 10 mg/kg (n=11) or both (n=11) by i.p. injection for 3 weeks after 9 days from tumor inoculation. Control mice were left untreated (n=11). Animal body weights were measured twice a week. 30 days after tumor inoculation, retro-orbital sampling was used to collect blood. And then, mice were euthanized with CO_2_ and tissues were isolated.

### Cell lines and culture

Human Burkitt’s lymphoma cell lines including Ramos (RRID: CVCL_0597) and Daudi (RRID: CVCL_0008), human B-cell lymphoma cell line RPMI1788 (RRID: CVCL_2710), human cutaneous T-cell lymphoma cell lines including H9 (RRID: CVCL_1240) and HuT78 (RRID: CVCL_0337), human Hodgkin’s lymphoma cell lines including Hs602 (RRID: CVCL_0815) and RPMI6666 (RRID: CVCL_1665) and were obtained from Korean Cell Line Bank (Seoul, Republic of Korea). These cell lines were grown in RPMI 1640 medium (#11875119, Gibco, Life Technologies, CA, USA) supplemented with 10–20% heat-inactivated fetal bovine serum (FBS; #26140079, Gibco), 1% GlutaMax (2 mM L-alanyl-L-glutamine; #35050-061, Gibco), 1% penicillin–streptomycin (Pen strep glutamine; #10378-016, Gibco). Except for HuT78, which was maintained in Dulbecco’s Modified Eagle Medium (#11965118, Gibco) supplemented with 10% heat-inactivated fetal bovine serum (FBS; #26140079, Gibco), 1% GlutaMax (2 mM L-alanyl-L-glutamine; #35050-061, Gibco), 1% penicillin–streptomycin (Pen strep glutamine; #10378-016, Gibco). All cell lines were incubated at 37°C in a humidified atmosphere containing 5% CO_2_.

### Isolation and activation of human CD8+T cells

Blood samples were collected from healthy donors after obtaining written informed consent [Institution Review Board (IRB) identifier INJE 2024-03-016-001]. CD8+ T cells were isolated from human whole peripheral blood mononuclear cells using Lymphoprep (#07801, StemCell Technologies, BC, Canada) according to the manufacturer’s instructions. For activation, 3 × 10^6^ CD8+ T cells were cultured in 6-well plates with plate-bound anti-CD3 (5 μg/mL, #16-0037-81, Invitrogen), anti-CD28 (5 μg/mL, #16-0289-81, Invitrogen) and recombinant human IL-2 (20 ng/mL, #RP-8608, Invitrogen) for 72-96 hours. Then, CD8+ T cells were harvested and co-cultured with human lymphoma cells for 24-72 hours. Cells were analyzed by cell proliferation assay and flow cytometry.

### Drug preparations

Tazemetostat (iEZH2, EZH2 inhibitor; #HY-13803) and MS1943 (dEZH2, EZH2 degrader; #HY-133129) were purchased from MedChemExpress (NJ, USA). These drugs were dissolved in dimethyl sulfoxide (DMSO; #1380, Duksan, Republic of Korea) as recommended by the manufacturer and stored at -80 °C. Pembrolizumab (aPD1, anti-PD-1; #SIM0010) was purchased from BioXCell (NH, USA) and stored at 4 °C. For *in vitro* studies, all of these drugs were diluted with RPMI 1640 medium with 10% heat-inactivated FBS, 1% GlutaMax (2 mM L-alanyl-L-glutamine; #35050-061, Gibco) and 1% penicillin–streptomycin (Pen strep glutemin; #10378-016, Gibco) before being used for the treatment of cell lines. For *in vivo* studies, MS1943 was dissolved in 40% PEG300, 5% Tween 80, and normal saline. Pembrolizumab was dissolved in normal saline.

### Cell proliferation assay

Cell viability was assessed using a WST-8 cell viability assay kit (#QM1000, Quanti-Max, Biomax, Republic of Korea). Human lymphoma cell lines were seeded at an initial cell density of 1 × 10^4^ cells/100 μL culture medium in 96-well plates. For co-culture experiments, each cell line was cocultured with activated human CD8+ T cells at an effector: target ratio of 5:1. Different doses of Tazemetostat and MS1943 drugs were administered to the cells, including concentrations of 2.5 μM, 5 μM, and 10 μM for each drug. Cells were treated as conditioned with 10 μg/mL Pembrolizumab and 10 ng/mL recombinant human IFN-γ (#300-02, PeproTech, USA). Additionally, cells were treated with combinations of these drugs or left untreated as controls. The cells were cultured for 72 hours. Then, 10 μL of WST-8 reagent was added to each well and the plates were incubated for 3 hours. Cultures were maintained at 37°C in a 5% CO_2_ atmosphere. Absorbance was measured at 450 nm using a microplate reader (SPECTROstar Nano, BMG Labtech, Germany). Cell viability is calculated by OD of test group/OD of control × 100%. All the experiments were performed at triplicate.

### Western blotting

Harvested cultured Ramos or Daudi cells were lysed in 2× Laemmli sample buffer (#BR1610747, Bio-Rad, Republic of Korea) with β-mercaptoethanol (#MR1015-100-00, Biosesang). NOG mice livers were minced and lysed in tissue extraction reagent 1 (#FNN0071, Invitrogen) containing protease inhibitor (#A32955, Thermo Scientific, MA, USA) and clarified by centrifugation (10,000×g for 5 min at 4°C). Protein concentrations were measured using a Bradford Protein Assay Kit (#BC1017-500- 02, Biosesang, Republic of Korea). Protein lysates (30 μg) were dissolved in 4× Laemmli sample buffer (#BR1610747, Bio-Rad) with β-mercaptoethanol (#MR1015-100-00, Biosesang). All samples were boiled at 95°C for 10 min. After removal of the insoluble fraction by centrifugation at 13,000 ×g for 10 min, protein samples were separated by SDS gel electrophoresis and transferred to a polyvinylidene difluoride membrane. Membranes were stained with EZH2 (#5246), c-MYC (#18583), PARP (#9542), cleaved caspase-3 (#9664), phospho-STAT1 (#9167) antibodies (Cell Signaling Technology, MA, USA) at a dilution of 1: 1,000 or GAPDH (#5174) antibody (Cell Signaling Technology) at a dilution of 1: 5,000 at 4°C overnight. After overnight incubation in 4°C, HRP-conjugated secondary antibody (#ADI-SAB-300-J, Enzo, NY, USA) was added. After washing with Tris-buffered saline (#CBT005, LPS Solution, Republic of Korea) and Tween 20(#TW2001, LPS Solution), the hybridized bands were detected using an enhanced chemiluminescence (ECL) detection kit (#K-12045-D50, Amersham Pharmacia Biotech, Buckinghamshire, UK).

### RNA extraction and cDNA synthesis

The 5 × 10^5^ cells treated with or without drugs same as described. Total RNA was extracted using TRIzol reagent (#15596018, Invitrogen) and the concentration of RNA was measured using the NanoDrop. These were reverse transcribed using High Capacity RNA-to-cDNA Kit (#4368813, Applied Biosystems, MA, USA) following to the manufacturer’s protocol. The cDNA was synthesized using Thermocycler (Bio-Rad, CA, USA) with cycling conditions used include primer annealing at 25°C for 10 min, DNA polymerization at 37°C for 120 min and finally reverse transcriptase deactivation at 85°C for 5 min. The synthesized cDNA was stored at -20°C before further use.

### Quantitative RT-PCR

qRT-PCR was performed in triplicate using a SYBR Green PCR Master Mix (#4309155, Applied Biosystems, MA, USA) using the manufacturer’s protocols: Hold 1 cycle for 120 sec at 50 °C and 30 sec at 95 °C, 2 step PCR 40 cycles for 15 sec at 95 °C and 60 sec at 60 °C. Add the melt curve steps consisting of 15 sec at 95 °C, 60 sec at 60 °C and 1 sec at 95 °C for 1 cycle. For detection the mechanisms of the treated drugs in Burkitt’s lymphoma, the following gene-specific primers were used: IRF-1 (forward: 5’-CATTCACACAGGCCGATACAAA-3’; reverse: 5’-AGCGAAAGTTGGCCTTCCA-3’), and GAPDH (forward: 5’-CCACTCCTCCACCTTTGACG-3’; reverse: 5’-CCACCACCCTGTTGCTGTAG -3’). For quantification, relative mRNA expression of specific genes was calculated using the 2-ΔΔCt method, after normalization to GAPDH expression.

### Cell cycle profiling and apoptosis assay

Ramos and Daudi cells were harvested and washed twice with FACS buffer (0.5% of FBS in PBS). The FITC Annexin V apoptosis detection kit with PI (#640914, BioLegend, CA, USA) for apoptosis analysis and PI (#421301) for cell cycle analysis were used following the recommended methods. The prepared samples were analyzed using a novocyte advanteon flow cytometer (Agilent, CA, USA).

### Flow cytometry

Ramos, Daudi and CD8+ T cell co-cultured lymphoma cells were harvested and washed twice with FACS buffer (0.5% of FBS in PBS). Then, the cells were resuspended in 100 μL of FACS buffer and stained with antibodies conjugated with fluorescein for 30 min at 4 °C. FITC HLA class 1 (#311404) and APC human CD274 (#329708) antibodies (BioLegend, CA, USA) were used to detect the expression of HLA class 1 and PD-L1. FITC Mouse IgG2a (#400207, BioLegend) and APC Mouse IgG2b (#400321, BioLegend) were used as an isotype control. The prepared samples were analyzed using Novocyte Advanteon flow cytometer (Agilent, CA, USA).

### ELISA

Concentrations of human IFN- γ in the plasma of humanized mice were measured with the Human IFN-γ Uncoated ELISA kit (#88-7316-88, Invitrogen) according to the manufacturer’s instructions.

### Immunofluorescence assay

Resected liver tissues and the spleen were fixed in 4% paraformaldehyde and embedded in paraffin. Paraffin-embedded blocks were sectioned at 4 μm. Sections were deparaffinized, rehydrated, and recovered antigen with proteinase K (#ab64220, Abcam, UK). Nonspecific background was blocked with 10% bovine serum albumin (#BSAS0.1, Bovogen, VIC, AU) in PBS for 60 min. For removal lipofuscin autofluorescence in tissues, TrueBlack (#23007, Biotium, CA, USA) was treated for 30 sec. Each tissue was stained with primary antibodies against EZH2 (#5246, Cell Signaling Technology), c-MYC (#18583, Cell Signaling Technology) CD8 (#MA1-81692, Invitrogen), granzyme B (#17215, Cell Signaling Technology), IFN-γ (#AF-285-NA, Novus Biologicals, CO, USA), and cleaved caspase-3 (#9664, Cell Signaling Technology) overnight at 4°C. Secondary antibodies, goat anti-rabbit IgG FITC (#31573, Invitrogen), goat anti-rabbit IgG Alexa Fluor 647 (#A21244, Invitrogen), donkey anti-goat IgG FITC (#bs-0294D-FITC, Bioss antibodies, MA, USA) and mouse anti-rat IgG, phycoerythrin (#bs-0293M-PE, Bioss antibodies, MA, USA), were applied for 90 min. Nuclei were counterstained with DAPI (#D1306, Invitrogen). Slides were mounted with fluorescent mounting medium (#S3023, Agilent Dako, CA, USA). For acquisition, data were acquired by sequential acquisition, and image was performed and analyzed on a highly sensitive confocal laser scanning microscope (#LSM800, Carl Zeiss, DEU) with ZEN microscopy software.

### Statistical analysis

Each experiment was repeated at least three times to ensure the reproducibility of the results. The results are expressed as mean ± SD. Statistical significance was determined using two-tailed unpaired Student’s t-test for multiple comparisons. Statistical analyses of all data were performed by GraphPad Prism (Version 8.0, GraphPad Software, Inc, San Diego, CA, USA). In all analyses, P values less than 0.05 were considered to indicate statistical significance.

## Results

### dEZH2 significantly reduces cell viability by inhibiting the expression of EZH2 and c-MYC in Burkitt’s lymphoma

**Figure 1:**
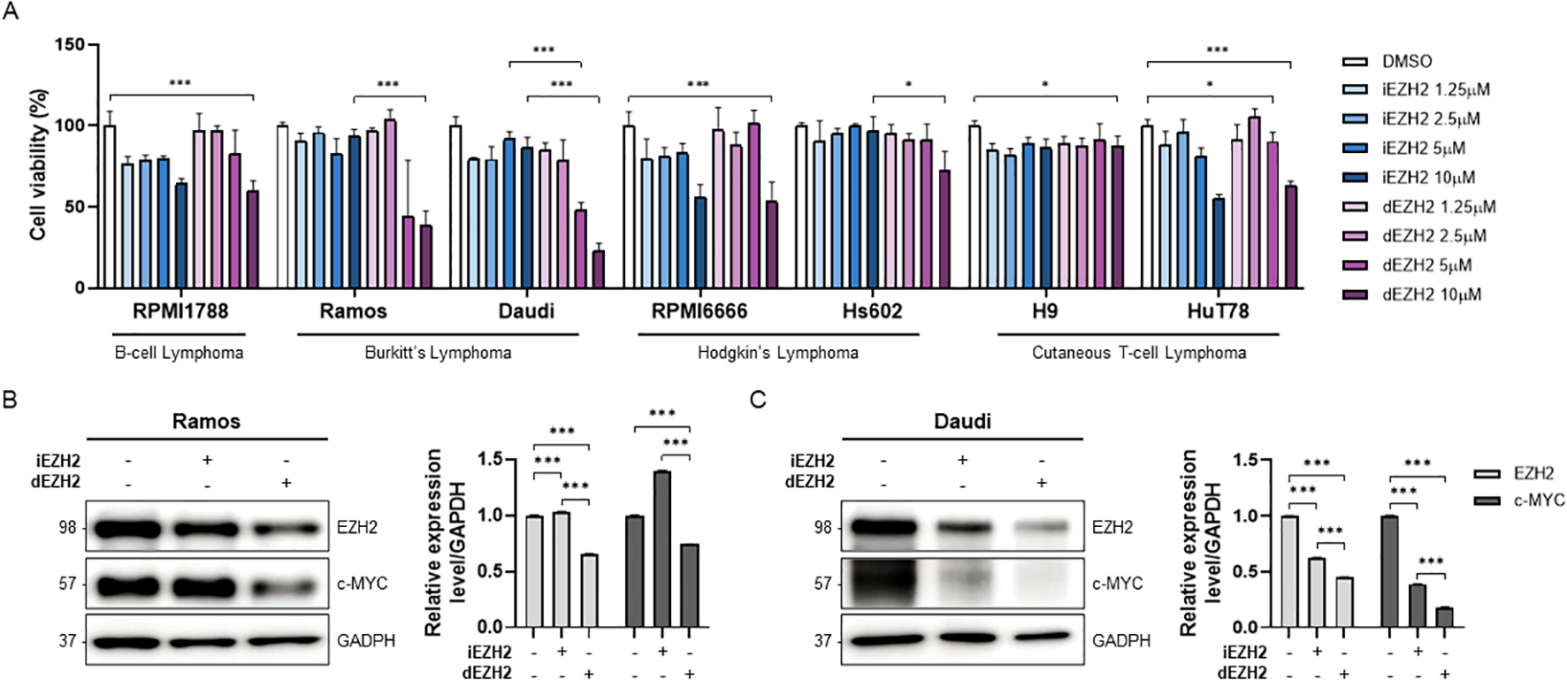
Dose-dependent effects of iEZH2 and dEZH2 in human lymphoma cell lines. **(A)** Human lymphoma cell lines were treated with 1.25 μM, 2.5 μM, 5 μM and 10 μM of iEZH2 or dEZH2 for 72 hours. Cell viability was compared with control group which was treated DMSO. **(B, C)** The protein expression level of EZH2 and c-MYC relative to GAPDH was measured by western blot analysis. Burkitt’s lymphoma cell lines, **(B)** Ramos and **(C)** Daudi, were exposed to 10 μM of iEHZ2 or dEZH2 for 24 hours. Error bars are shown as mean ± SD. Data analyzed using two-tailed unpaired student’s t-tests. *, P ≤ 0.05; ***, P ≤ 0.001.

First, we conducted a proliferation assay to compare the effects of the EZH2 inhibitor (iEZH2, Tazemetostat) and degrader (dEZH2, MS1943) on cell viability across various lymphoma cell lines. We cultured nine lymphoma cell lines, including B-cell lymphoma, Burkitt’s lymphoma, Hodgkin’s lymphoma, and Cutaneous T-cell lymphoma, with increasing concentrations of iEZH2 or dEZH2 for 72 hours. Interestingly, we observed that while iEZH2 showed non-effectiveness in Ramos and Daudi cell lines, which are Burkitt’s lymphoma, treatment with 5 and 10 µM of dEZH2 resulted in a significant decrease in cell viability. In other cell lines, both iEZH2 and dEZH2 exhibited negligible inhibitory effects, with over 50% cell survival rates observed (**Figure 1A**).

Next, we performed a western blot analysis to confirm whether iEZH2 and dEZH2 actually reduce EZH2 expression levels in Burkitt’s lymphoma cells. We noted a distinct reduction in EZH2 expression with dEZH2 compared to iEZH2 in both Ramos and Daudi cells. Additionally, we assessed the expression levels of c-MYC, a gene known to be overexpressed in Burkitt’s lymphoma and recognized as an important therapeutic target. In each of the cell lines, we confirmed that the c-MYC levels were decreased with dEZH2 treatment (**Figures 1B, C**). Our results provide evidence that the use of dEZH2 could represent a significant therapeutic strategy in Burkitt’s lymphoma.

### Enhanced apoptosis and cell cycle arrest are caused by dEZH2 in Burkitt’s lymphoma

**Figure 2:**
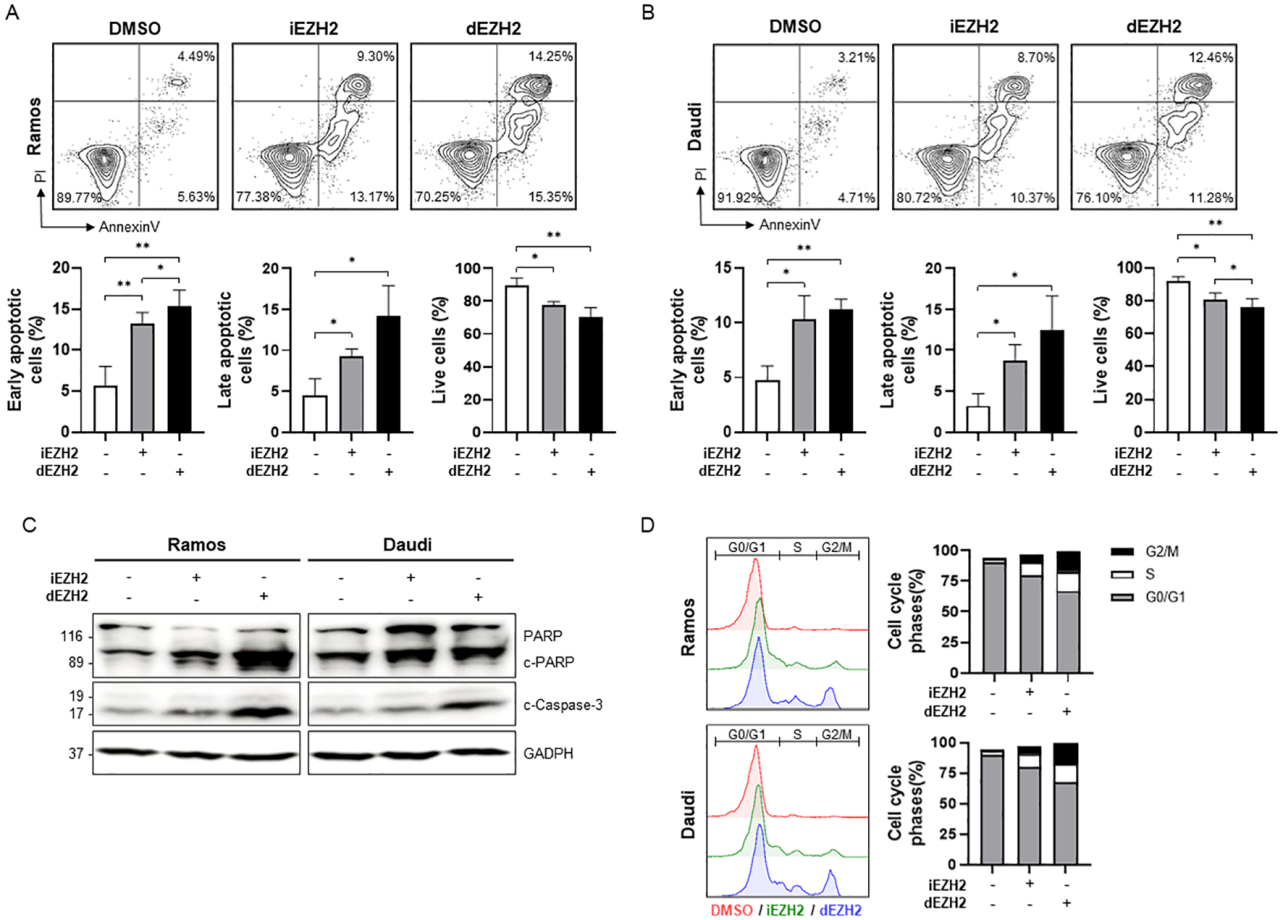
Upregulation of apoptosis and G2/M phase arrest caused by dEZH2 in Burkitt’s lymphoma. **(A, B)** Annexin V/PI staining was performed to determine apoptotic effect in **(A)** Ramos and **(B)** Daudi by flow cytometry. Ramos and Daudi cells were treated with 10 μM of iEHZ2 or dEZH2 for 72 hours. The percentages of early apoptotic cells (Annexin V^+^/PI^−^), late apoptotic cells (Annexin V^+^/PI^+^) and live cells (Annexin V^−^/PI^−^) were expressed in sequence from left to right in the graphs for each cell line. **(C)** The western blot assay was used to analyze apoptosis-associated proteins, including PARP, cleaved PARP (c-PARP) and cleaved caspase-3 (c-Caspase-3) in Ramos and Daudi cell lines. Protein levels were evaluated after 72 hours of treatment with 10 μM of iEHZ2 or dEZH2. GAPDH was utilized for normalization. **(D)** The cell cycle arrest was detected by flow cytometry with PI staining. Ramos and Daudi cells were treated with 10 μM of iEHZ2 or dEZH2 for 72 hours. Error bars are shown as mean ± SD. Data analyzed using two-tailed unpaired student’s t-tests. *, P ≤ 0.05; **, P ≤ 0.01.

To further investigate whether the inhibition of EZH2 induces cell death in Burkitt’s lymphoma, we performed flow cytometry using Annexin V/PI staining. We observed a significant increase in both early and late apoptotic cells following dEZH2 treatment in both Ramos and Daudi cell lines, while the proportion of live cells was relatively decreased (**Figures 2A, B**). To support these findings, we confirmed the expression of well-known apoptosis-associated proteins, including PARP, cleaved PARP, and cleaved caspase-3, through western blot analysis. Both cleaved PARP and cleaved caspase-3 exhibited markedly increased expression levels under dEZH2 treatment, consistent with the results from flow cytometry (**Figure 2C**). Also, we investigated the relationship between cell death and cell cycle arrest by analyzing the cell cycle distribution. Upon treatment with dEZH2, we confirmed an increase in the G2/M phase in both Ramos and Daudi cell lines (**Figure 2D**). Our results show that dEZH2 suppresses cell viability in Burkitt’s lymphoma through the induction of apoptosis and cell cycle arrest.

### dEZH2 promotes the expression of HLA class 1 in the presence of IFN-γ, while PD-L1 expression is induced independently of IFN-γ *in vitro*

**Figure 3:**
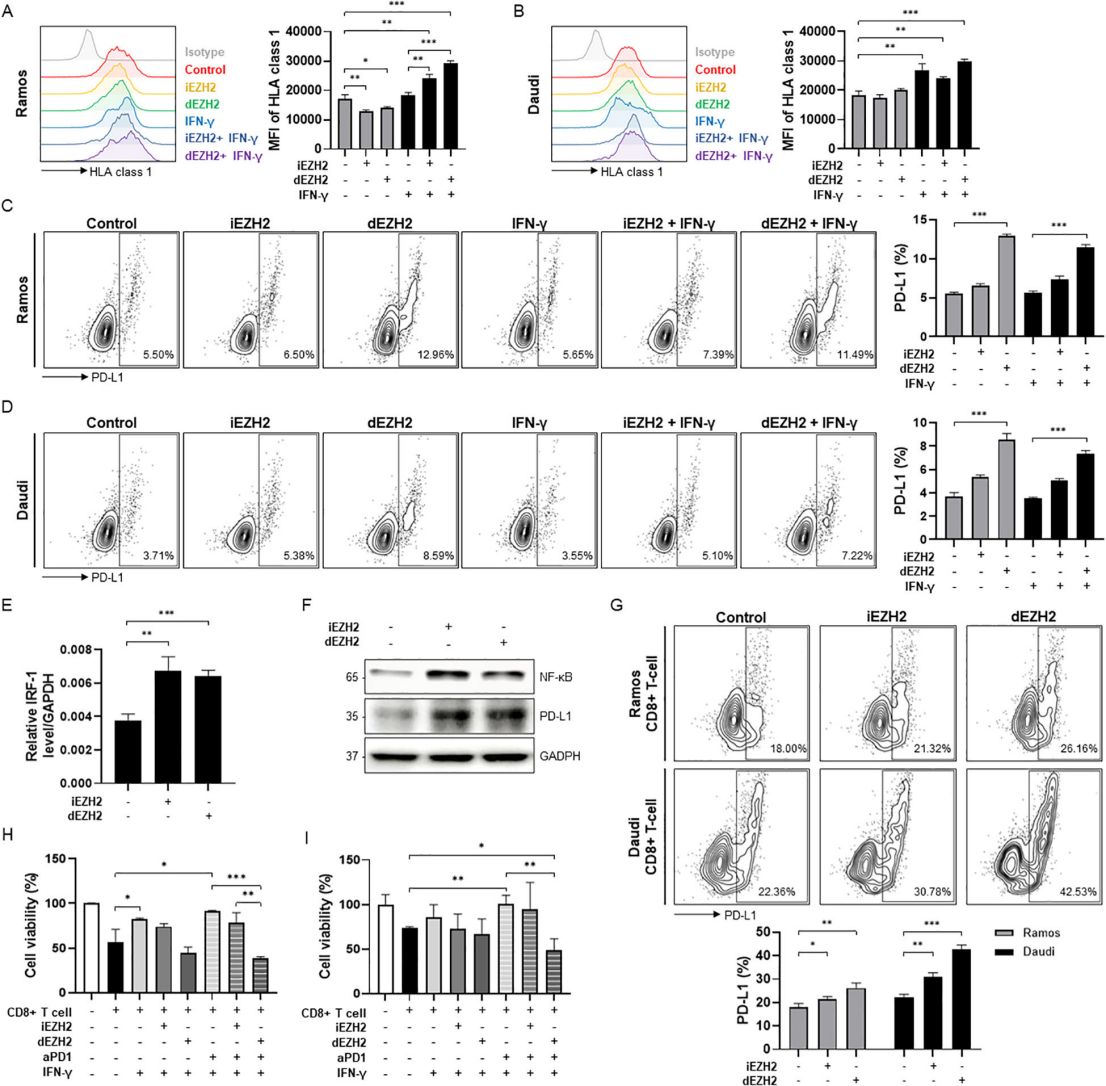
Enhanced levels of HLA class 1 and PD-L1 affected by EZH2 inhibition or IFN-γ in Burkitt’s lymphoma. **(A, B)** HLA class 1 expression levels were measured by flow cytometry. **(A)** Ramos and **(B)** Daudi cells were exposed to 10 μM of iEHZ2 or dEZH2, along with 10 ng/mL of human IFN-γ for 48 hours. Compared to the isotype control, HLA class 1 levels were analyzed using histogram and are shown in the graphs on the right. **(C, D)** Expression levels of PD-L1 were determined using flow cytometry with dot plots. PD-L1 levels were compared to the isotype control, and the analyzed data are presented in the graphs below. 10 ng/mL of human IFN-γ was treated in **(C)** Ramos and **(D)** Daudi cell lines for 48 hours, along with 10 μM of iEHZ2 or dEZH2. **(E)** The relative IRF-1 mRNA expression in Daudi was measured by RT-qPCR, and normalized to GAPDH. Daudi cells were exposed to 10 μM of iEHZ2 or dEZH2 for 24 hours. **(F)** The expression levels of NF-κB and PD-L1 proteins, which were compared to GAPDH, were detected by western blot analysis. Daudi cells were treated with 10 μM of iEHZ2 or dEZH2 for 48 hours. **(G)** Ramos and Daudi cells were plated with *in vitro* activated CD8+ T cells at an E:T ratio of 1:1. Co-cultured cells were treated with 10 nM iEZH2 or dEZH2. After 48 hours, PD-L1 levels were measured by flow cytometry. **(H, I)** Activated CD8+ T cells co-cultured **(H)** Ramos and **(I)** Daudi cells (E:T ratio of 5:1) were treated by 10 μg/mL aPD1 with 10 nM dEZH2 or iEHZ2 for 72 hours in the presence IFN-γ. Cell viability was determined by WST-8 assay. Error bars are shown as mean ± SD. Data analyzed using two-tailed unpaired student’s t-tests. *P ≤ 0.05; **P ≤ 0.01; ***P ≤ 0.001.

Next, we investigated the role of EZH2 inhibition in immune evasion in Burkitt’s lymphoma. We treated the Ramos and Daudi cells with 10 µM of iEZH2 or dEZH2, assessing the expression levels of HLA class I through flow cytometry under conditions with and without IFN-γ. In both Ramos and Daudi cell lines, we observed no significant differences in expression levels between iEZH2 and dEZH2; however, a slight increase was noted in the presence of IFN-γ (**Figures 3A, B**).

Additionally, we evaluated the expression levels of PD-L1 under the same conditions. PD-L1 binds to PD-1 on T-cells, thereby inducing immune evasion. Thus, inhibiting this interaction is crucial for promoting the death of cancer cells in therapeutic settings (22). Our results indicated that in both cell lines treated with dEZH2, the expression of PD-L1 increased slightly compared to the control (**Figures 3C, D**). Also, to investigate the mechanism of PD-L1 expression caused by EZH2 inhibition, expression levels of PD-L1 promoters, such as NF-κB, STAT-1, STAT-3 and IRF-1, were measured by western blot or qRT-PCR. We observed that NF-κB and IRF-1 were upregulation upon EZH2 inhibition (**Figures 3E, F**). Our findings highlight that treatment with dEZH2 induces PD-L1 expression in PD-L1-negative Burkitt’s lymphoma, mediated by increased levels of NF-κB and IRF-1.

### The combination of dEZH2 and aPD-1 in CTL co-cultured Burkitt’s lymphoma cells inhibited cell proliferation *in vitro*

The above findings indicate that the use of EZH2 inhibition in Burkitt’s lymphoma can facilitate immune evasion, thereby maintaining the survival of cancer cells within the human body. We specifically investigated how the inhibition of EZH2 affects PD-L1 expression and cell viability in the presence of immune cells. First, we treated Burkitt’s lymphoma cell lines with 10 µM of either iEZH2 or dEZH2 in a co-culture environment with cytotoxic T lymphocytes (CTLs; CD8+ T cells) and assessed PD-L1 expression through flow cytometry. In both cell lines, we observed an increase in PD-L1 expression upon EZH2 inhibition, with a more pronounced increase noted following dEZH2 treatment (**Figure 3G**). Based on these findings, we hypothesized that in order to enhance the therapeutic efficacy of EZH2 inhibition in Burkitt’s lymphoma within an immune cell-rich environment, it is crucial to limit the activity of PD-L1 using anti-PD-1 (aPD1) treatment. We co-treated CTL co-cultured Burkitt’s lymphoma cells with aPD1 alongside iEZH2 or dEZH2, and performed a proliferation assay to investigate cell viability in an environment supplemented with IFN-γ to promote immune activity. Our results demonstrated that the combination treatment of dEZH2 and aPD1 yielded the highest therapeutic efficacy compared to the individual treatments (**Figures 3H, I**). This highlights that combination of dEZH2 and aPD1 effectively induces cancer cell death in Burkitt’s lymphoma within an immune cell-rich environment.

### Combination therapy reduces Burkitt’s lymphoma by decreasing EZH2 levels in hu-PBMC-CDX mouse model

**Figure 4:**
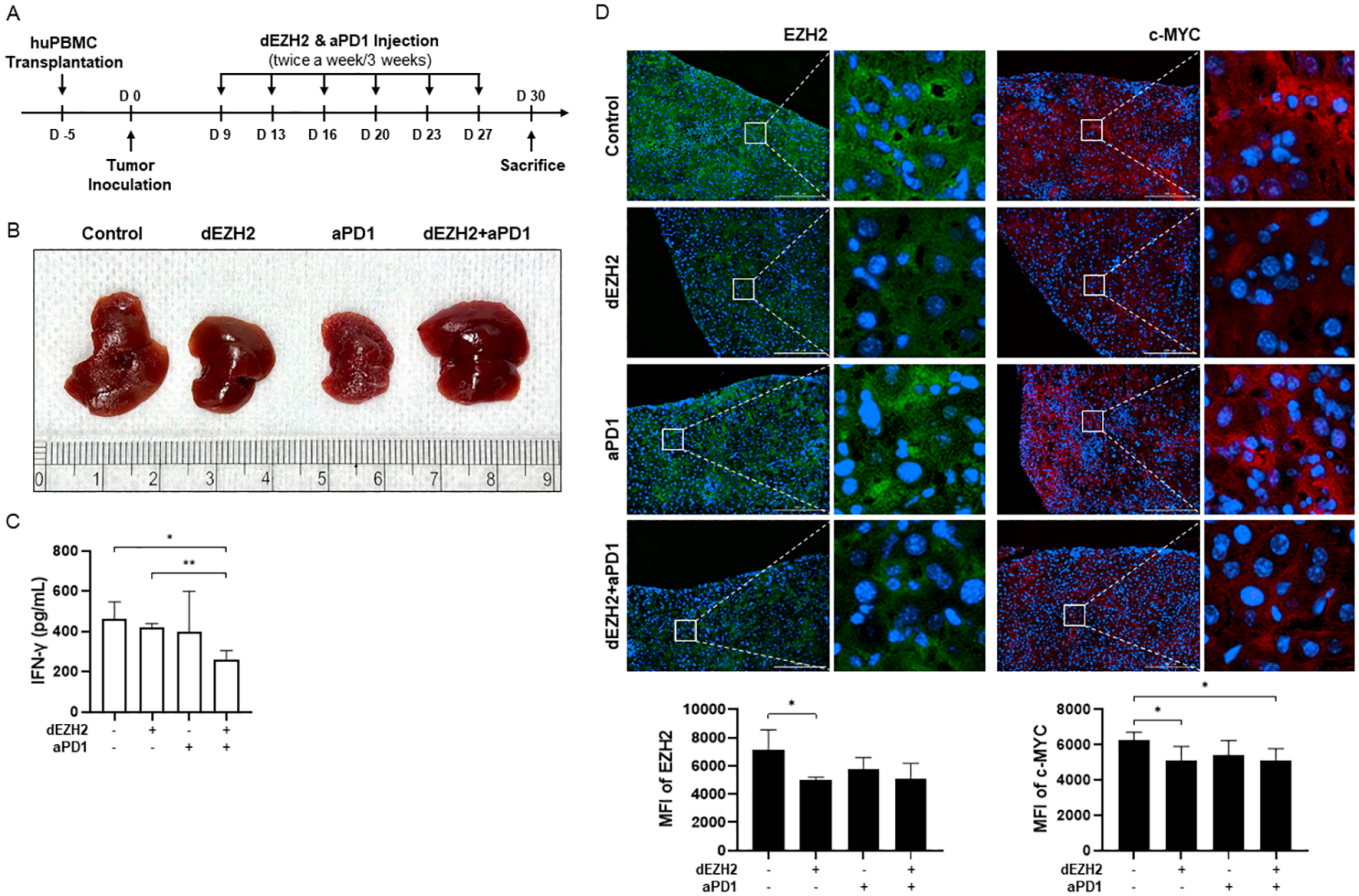
Effect of dEZH2 and aPD1 treatment on Burkitt’s lymphoma growth and EZH2 expression. **(A)** Experimental timeline of the *in vivo* injection for combination therapy. **(B)** The liver of each treatment or control group was harvested, photographed at the end of treatment. **(C)** Plasma was collected at the end of treatment. IFN-γ concentration was measured by ELISA. **(D)** EZH2 (green) and c-MYC (red) expressions in the liver were indicated on the immunofluorescence images. Mean fluorescence intensity (MFI) of EZH2 and c-MYC were shown in the graph. Scale bar = 200 µm. Error bars are shown as mean ± SD. Data analyzed using two-tailed unpaired student’s t-tests. * P ≤ 0.05; ** P ≤ 0.01.

Based on previous *in vitro* studies, we conducted an *in vivo* study using a human PBMC-transplanted cell line-derived xenograft (hu-PBMC-CDX) model in immunodeficient NOG mice to closely examine interactions with immune cells. Humanization was achieved by inoculating NOG mice with human PBMCs, and analyses were performed on those exhibiting more than 25% huCD45+CD3+ T cells (data not shown). Five days post-PBMC inoculation, the human Burkitt’s lymphoma cell line, Daudi, was introduced to observe tumor progression. Mice were subsequently treated intravenously with dEZH2 (50 mg/kg) and/or aPD1 (10 mg/kg), administered twice weekly for a total of three weeks. On 30 days post-tumor injection, the mice were euthanized, and plasma, liver, and spleen samples were collected for subsequent analysis (**Figure 4A**). First, tumor morphology in the liver was compared, as Burkitt’s lymphoma typically exhibits a disseminated growth pattern rather than forming solid tumors in organs. Unlike the smooth surface of a normal liver, tumor infiltration leads to a rough and nodular appearance, reflecting the extent of disease progression. The tumor progression was notably better in the dEZH2 and combination treatment groups compared to other groups (**Figure 4B**). Additionally, IFN-γ concentrations in mouse plasma showed a slight decrease in the combination-treated group relative to the control (**Figure 4C**). Furthermore, expression levels of EZH2 and c-MYC in liver tissue were reduced in the dEZH2-treated group compared to control, suggesting that dEZH2 maintains EZH2 degradation activity *in vivo* and effectively inhibits the progression of Burkitt’s lymphoma (**Figure 4D**). Our results demonstrate that the efficacy of dEZH2 with or without aPD1 in promoting EZH2 degradation and inhibiting tumor proliferation is also evident in a mouse model.

### Cell apoptosis is induced combination treatment with dEZH2 and aPD1 through activated CD8+ T cell in Burkitt’s lymphoma

**Figure 5:**
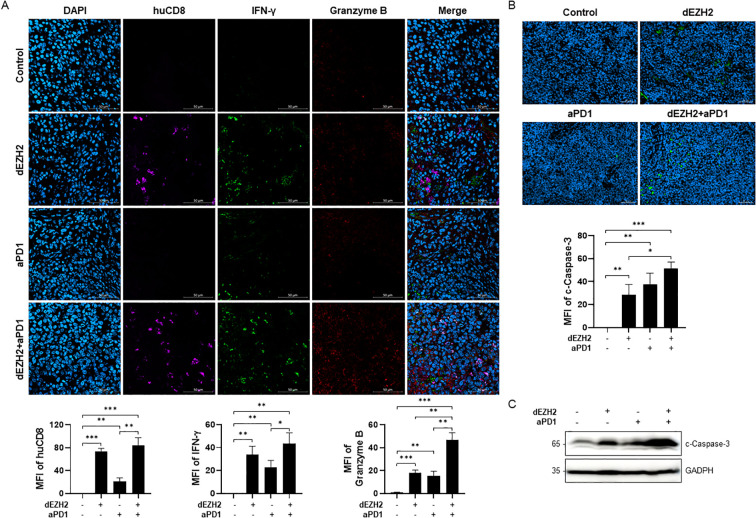
Upregulation of apoptosis via CD8+ T cells in Burkitt’s lymphoma mouse model. **(A)** Immunofluorescence images of spleen sections from each group were exposed huCD8 (violet), IFN-γ (green) and granzyme B (red). MFI of each target was expressed in the graph. Scale bar = 200 µm. **(B)** Cleaved caspase-3 on spleen in each indicated condition was analyzed by immunofluorescence staining. MFI values were shown in the graph on the bottom. Scale bar = 50 µm. **(C)** Expression level of cleaved caspase-3 in liver was measured by western blot. Error bars are shown as mean ± SD. Data analyzed using two-tailed unpaired student’s t-tests. *P ≤ 0.05; **P ≤ 0.01; ***P ≤ 0.001.

Next, to evaluate CD8+ T cell activation within tumor in the hu-PBMC-CDX mouse model, we assessed CD8+ T cell infiltration in the liver and spleen tissues through immunofluorescence. Significantly higher infiltration of human CD8+ T cells was observed in the tissues and tumors of the dEZH2 and aPD1 combination group compared to the control group. Additionally, IFN-γ and granzyme B, cytokines secreted by activated CD8+ T cells, were elevated in the combination group (**Figure 5A**).

Granzyme B, a cytotoxic molecule secreted by CD8+ T cells, plays a critical role in eliminating virus-infected cells and tumors. Upon entering the cytoplasm, granzyme B activates various proteins, particularly caspase-3, thereby inducing apoptosis (23). Based on prior findings of dEZH2-induced apoptosis in cell line studies, we investigated apoptotic activity in the mouse model by assessing the expression of cleaved caspase-3. Immunofluorescence analysis of spleen tissues demonstrated that cleaved caspase-3 expression was significantly higher in the combination treatment group compared to other groups (**Figure 5B**). Similarly, western blot analysis of liver tissues revealed the highest levels of cleaved caspase-3 in the combination-treated group (**Figure 5C**). Our results indicate that the combination of dEZH2 and aPD1 enhances CD8+ T cell activation and induces significant anti-tumor effects by promoting tumor cell apoptosis *in vivo*.

## Discussion

Burkitt’s lymphoma is a highly aggressive hematologic malignancy of B-cells with a high proliferation rate (24). Burkitt’s lymphoma is characterized by the overexpression of c-MYC, which is implicated in the pathogenesis of Burkitt’s lymphoma and other lymphoma subtypes. MYC is a crucial transcription factor that drives the overexpression of EZH2 (25), and previous study has shown that 80-100% of Burkitt’s lymphoma cells exhibit positive MYC expression. This finding supports this correlation with high EZH2 expression in Burkitt’s lymphoma (14). EZH2 and c-MYC are involved in the regulation of apoptosis and cell cycle progression. Therefore, targeting EZH2 represents a potential therapeutic strategy for treating Burkitt’s lymphoma (3, 26).

In this study, MS1943, a PROTAC-based EZH2 degrader, demonstrated notable efficacy in suppressing human lymphoma cell lines, especially Burkitt’s lymphoma, compared to the conventional EZH2 inhibitor, Tazemetostat (**Figure 1A**). Also, MS1943 demonstrated exceptional degradation activity against EZH2 and c-MYC (**Figures 1B, C**). These findings suggest that targeting EZH2 is proposed as an effective therapeutic strategy for treating Burkitt’s lymphoma. Furthermore, compared to EZH2 inhibitor, the degrader exhibited significantly higher levels of G2/M phase arrest and an enhanced induction of apoptosis (**Figures 2A–D**). These results indicate that EZH2 degrader serves as more effective therapeutic agents for the treatment of Burkitt’s lymphoma than conventional EZH2 inhibitor.

In addition, our study investigated the anti-tumor effect induced by the interaction of immune cells within mouse model when simultaneously treating with the EZH2 degrader and the immune checkpoint inhibitor, anti-PD-1, and elucidating the underlying mechanisms. Immune checkpoints serve as critical regulators of the immune system, facilitating immune self-tolerance and preventing uncontrolled immune responses. However, in certain malignancies, these immune checkpoints can be aberrantly activated, enabling tumors to evade immune surveillance and resist immune-mediated attacks. One primary strategy involves utilizing mechanisms that negatively regulate CTLs function. Immune checkpoint proteins such as programmed cell death protein 1 (PD-1), PD-1 ligand 1 (PD-L1), and cytotoxic T-lymphocyte-associated antigen 4 (CTLA-4) inhibit CTL immune responses. When PD-L1 expressed on cancer cells binds to PD-1 on CTLs, it limits the anti-cancer activity of the CTLs, and cancer cells can upregulate PD-L1 expression to evade immune detection (27, 28). Immune checkpoint inhibitors have resulted in significant clinical success in cancer therapy; however, not all patients respond to these treatments (29, 30). Therefore, it is essential to develop treatment strategies that combine immune checkpoint inhibitor with other immunotherapies or targeted therapies. Additionally, role of EZH2 in the epigenetic regulation of gene expression is recognized as a crucial element in influencing immune evasion in cancer cells (21). In lung cancer cells, EZH2 has been found to induce the activity of hypoxia-inducible factor 1α, thereby promoting the expression of PD-L1, establishing a positive correlation (31). However, in hepatocellular carcinoma cells, EZH2 enhances H3K27me3 at the promoters of CD274, which encodes PD-L1, and interferon regulatory factor 1 (IRF1), leading to an inverse correlation by inhibiting PD-L1 expression (32). Burkitt’s lymphoma is also characterized by the overexpression of EZH2. And also, previous studies have shown that, dissimilar to other lymphomas where PD-L1 is overexpressed, the expression of PD-L1 is often lower or occasionally absent in Burkitt’s lymphoma (33, 34). However, the role of EZH2 in PD-L1 expression in Burkitt’s lymphoma has not been investigated.

We observed an increase in PD-L1 expression with EZH2 inhibition, regardless of the presence of immune cells although Burkitt’s lymphoma is a PD-L1 negative tumor (**Figures 3C, D, G**). The increased expression of promoters of PD-L1, such as NF-κB and IRF-1 through qRT-PCR analysis and western blot, demonstrated this finding (**Figures 3E, F**). In contrast, the expression of HLA class 1, which is critical for immune cell recognition of cancer cells, was slightly elevated only in the presence of IFN-γ, and was not affected by EZH2 inhibition (**Figures 3A, B**). These suggest that in the treatment of Burkitt lymphoma through EZH2 degradation, tumor cells could evade immune responses mediated by CTLs to enhance their survival. Therefore, the combination of EZH2 degraders with immune checkpoint inhibitors is essential for achieving effective therapeutic outcomes.

In an *in vivo* study, the liver of hu-PBMC-CDX mouse showed less tumor cell infiltration compared to the control (**Figure 4B**), along with decreased expression levels of EZH2 and c-MYC, indicating that dEZH2 effectively suppresses Burkitt’s lymphoma (**Figure 4D**). These results indicate that EZH2 degraders maintain their efficacy to degrade EZH2 and suppress the survival of Burkitt’s lymphoma cells *in vivo*. Furthermore, graft-versus-host disease (GVHD) is a severe systemic disorder caused by the donor immune cells attacking the host’s tissues and cells. The graft-versus-lymphoma (GVL) effect is associated with GVHD, contributing to anti-tumor responses and reduction of the tumor relapse. Both GVHD and GVL are associated with T-cell activity, and numerous studies are currently exploring strategies to inhibit GVHD while enhancing GVL responses (35, 36). In this study, GVHD and GVL effects were monitored in mice, with lower IFN-γ levels in the combination treatment group suggesting the potential for a GVL effect with reduced GVHD (**Figure 4C**). Moreover, the significant infiltration of CD8+ T cells and increased release of IFN-γ and granzyme B in liver and spleen provide evidence of enhanced anti-tumor activity by CD8+ T cells, compared to control group (**Figure 5A**). Additionally, the elevated expression level of cleaved caspase-3 demonstrated the significantly enhancement of apoptosis mediated by the anti-tumor effects of immune cells and dEZH2 treatment (**Figures 5B, C**).

**Figure 6:**
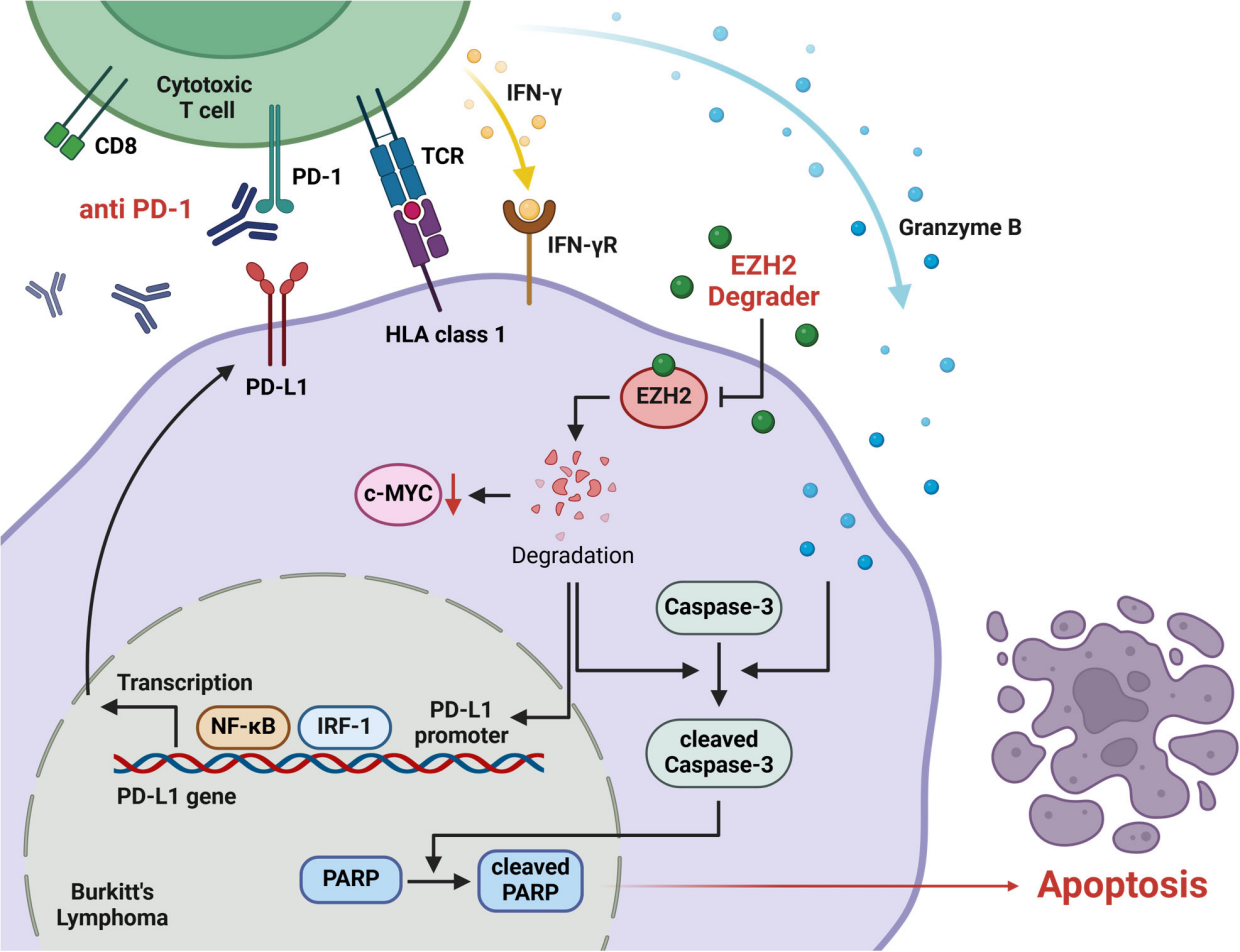
The signaling pathway of anti-PD-1 and EZH2 degradation that induces apoptosis in Burkitt’s lymphoma. EZH2 degrader suppresses the expression of c-MYC, a key oncogene in Burkitt’s lymphoma. Also, this degradation promotes the transcriptional regulation of PD-L1 through NF-kB and IRF-1, which regulate PD-L1 expression on the tumor cell surface. The anti-PD-1 inhibits the PD-1/PD-L1 immune checkpoint pathway, effectively preventing the PD-L1 expressed on tumor cells from binding to PD-1 on the CD8+ T cell. This inhibition restores T cell activation, thereby enabling the release of cytotoxic molecules, such as IFN-γ and granzyme B. The increased level of granzyme B and EZH2 degradation activates apoptotic pathways, particularly by promoting the activation of caspase-3. Cleavage of caspase-3 and PARP signifies the induction of apoptosis. The combination of anti-PD-1 and EZH2 degradation amplifies the immune response while directly targeting apoptotic pathways, effectively inducing apoptosis in Burkitt’s lymphoma.

In conclusion, our study demonstrated that combining dEZH2 and aPD1 in Burkitt’s lymphoma counteracted immune evasion caused by EZH2 inhibition-induced PD-L1 upregulation, leading to significantly enhanced apoptosis both *in vitro* and *in vivo* (**Figure 6**). Based on our findings, we propose that the combination of an EZH2 degrader and anti-PD-1 therapy may serve as an effective treatment strategy for patients with Burkitt’s lymphoma. Further studies involving a broader range of hematologic malignancies and solid tumor cells are needed to investigate PD-L1 expressions in the context of EZH2 degradation and to validate our findings. Moreover, the establishment of therapeutic efficacy requires the additional preclinical and clinical Burkitt lymphoma models, including patient-derived xenograft (PDX) models.

## Data Availability

The original contributions presented in the study are included in the article/supplementary material. Further inquiries can be directed to the corresponding author.
